# Identification of a dysfunctional exon-skipping splice variant in *GLUT9*/*SLC2A9* causal for renal hypouricemia type 2

**DOI:** 10.3389/fgene.2022.1048330

**Published:** 2023-01-17

**Authors:** Yu Toyoda, Sung Kweon Cho, Velibor Tasic, Kateřina Pavelcová, Jana Bohatá, Hiroshi Suzuki, Victor A. David, Jaeho Yoon, Anna Pallaiova, Jana Šaligová, Darryl Nousome, Raul Cachau, Cheryl A. Winkler, Tappei Takada, Blanka Stibůrková

**Affiliations:** ^1^ Department of Pharmacy, The University of Tokyo Hospital, Tokyo, Japan; ^2^ Molecular Genetics Epidemiology Section, Basic Research Laboratory, National Cancer Institute and Frederick National Laboratory for Cancer Research, Frederick, MD, United States; ^3^ Department of Pharmacology, Ajou University School of Medicine, Suwon, South Korea; ^4^ Faculty of Medicine, University Ss. Cyril and Methodius, Skopje, North Macedonia; ^5^ Institute of Rheumatology, Prague, Czechia; ^6^ Cancer and Developmental Biology Laboratory, Center for Cancer Research, National Cancer Institute, Frederick, MD, United States; ^7^ Nephro Dialysis Center, Michalovce, Slovakia; ^8^ Metabolic Clinic, Children’s Faculty Hospital, Košice, Slovakia; ^9^ CCR Collaborative Bioinformatics Resource, Center for Cancer Research, National Cancer Institute, Frederick, MD, United States; ^10^ Integrated Data Science Section, Research Technologies Branch, National Institute of Allergies and Infectious Diseases, Bethesda, MD, United States; ^11^ Department of Rheumatology, First Faculty of Medicine, Charles University, Prague, Czechia; ^12^ Department of Pediatrics and Inherited Metabolic Disorders, First Faculty of Medicine, Charles University and General University Hospital, Prague, Czechia

**Keywords:** genetic disorder, renal urate handling, RHUC, splicing variant, urate

## Abstract

Renal hypouricemia (RHUC) is a pathological condition characterized by extremely low serum urate and overexcretion of urate in the kidney; this inheritable disorder is classified into type 1 and type 2 based on causative genes encoding physiologically-important urate transporters, *URAT1* and *GLUT9*, respectively; however, research on RHUC type 2 is still behind type 1. We herein describe a typical familial case of RHUC type 2 found in a Slovak family with severe hypouricemia and hyperuricosuria. *Via* clinico-genetic analyses including whole exome sequencing and *in vitro* functional assays, we identified an intronic *GLUT9* variant, c.1419+1G>A, as the causal mutation that could lead the expression of p.Gly431GlufsTer28, a functionally-null variant resulting from exon 11 skipping. The causal relationship was also confirmed in another unrelated Macedonian family with mild hypouricemia. Accordingly, non-coding regions should be also kept in mind during genetic diagnosis for hypouricemia. Our findings provide a better pathogenic understanding of RHUC and pathophysiological importance of GLUT9.

## 1 Introduction

Uric acid is the end product of purine metabolism in humans and predominantly exists as urate in physiological conditions. The increasing global prevalence of urate-related diseases including hyperuricemia and gout (Dehlin et al., 2020; Safiri et al., 2020) has attracted interest to serum urate lowering; however, extremely low serum urate (sU)—hypouricemia (sU ≤ 120 μM, 2 mg/dL) (Nakayama et al., 2019; Nakayama et al., 2021)—has been also recognized as a pathological condition caused by decreased uric acid production (purine metabolism abnormality) or increased renal urate elimination (decreased renal urate re-uptake) (Pineda et al., 2020; Otani et al., 2022). In the latter case, hypouricemic condition is termed as renal hypouricemia (RHUC), which can be accompanied by exercise-induced acute kidney injury and kidney stones (Yeun and Hasbargen, 1995; Nakayama et al., 2019; Hosoyamada, 2021).

Renal hypouricemia is currently classified into two types according to its causative genes—genetic dysfunction in *urate transporter 1* (*URAT1*, also known as *SLC22A12*) and *glucose transporter 9* (*GLUT9*, also known as *SLC2A9*) corresponds to RHUC type 1 (Enomoto et al., 2002; Ichida et al., 2004; Iwai et al., 2004; Cheong et al., 2005; Wakida et al., 2005) and RHUC type 2 (Anzai et al., 2008; Matsuo et al., 2008; Dinour et al., 2010; Stiburkova et al., 2011; Stiburkova et al., 2012), respectively. URAT1 and GLUT9 are physiologically-important urate transporters involved in the re-absorption of urate from urine to blood in renal tubular cells as apical (urine side) and basal (blood side) machineries, respectively (Halperin Kuhns and Woodward, 2021). While familial RHUC is a relatively rare disorder in globally, its prevalence is reportedly higher in certain populations, including the Japanese (Nakayama et al., 2019; Koto et al., 2022), Korean (Son et al., 2016), and European Roma (Stiburkova et al., 2016), than in other populations. Nevertheless, most cases of reported RHUC were related with *URAT1* dysfunction, meaning that RHUC type 1 is the dominant type (Nakayama et al., 2022). For this reason, fewer studies have been reported for RHUC type 2 compared with RHUC type 1. Further investigations on RHUC type 2 are warranted for a better understanding of its genetic aetiology.

According to a recent study (Kawamura et al., 2021), patients with RHUC type 2 tend to exhibit higher values of fractional excretion of uric acid (FE_UA_, an indicator for the ratio of urinary-excreted urate/filtered urate by glomeruli) compared with RHUC type 1, suggesting a strong relationship between severe RHUC phenotypes and harboring homozygous or compound heterozygous dysfunctional variants in *GLUT9*. However, only a few causal variants in *GLUT9* for RHUC type 2 have been previously identified and all have been exonic non-synonymous mutations with the sole exception of one case with a 36-kb deletion containing exon 7 of *GLUT9* (Dinour et al., 2010). Intronic variations in *GLUT9* also have been identified as serum urate-affecting factors *via* genome-wide association studies focused on hyperuricemic phenotypes (Doring et al., 2008; Vitart et al., 2008; Major et al., 2018). In this context, addressing intronic regions of *GLUT9* should also be important to explore causative variations for RHUC of which genetic causes remain unknown.

Hitherto, we have enrolled dysuricemia (hyperuricemia and hypouricemia) individuals from the Roma populations (Stiburkova et al., 2016; Toyoda et al., 2019; Pavelcova et al., 2020; Stiburkova et al., 2021). In this process, we serendipitously found a family with RHUC of which the proband exhibited severe phenotypes; however, its causality could not be explained by already-characterized genetic variations responsible for RHUC, as described below. For this reason, to identify genetic factor(s) responsible for this familial RHUC, we herein conducted more comprehensive analyses covering non-coding genomic regions.

In this study, *via* clinico-genetic analyses, we identified a causative intronic variant—*GLUT9* c.1419+1G>A—in the family with RHUC. The causal relationship was also supported by the results of another Macedonian family with mild hypouricemia; genetic unrelatedness between the families were confirmed by whole exome sequencing (WES). This mutation was suggested to be resulted in a splicing error producing a frameshift variant of GLUT9 according to a theory (Anna and Monika, 2018). *Via* biochemical and functional analyses, we identified this variant as functionally null. These findings will deepen our understanding of RHUC type 2 as well as physiological impact of GLUT9 as a renal urate transporter.

## 2 Methods

### 2.1 Ethics approval

This study including human participants was approved by the Ethics Committee of the Institute of Rheumatology in Prague (No. 6181/2015) and the Ethical Commission of Medical Faculty Skopje, University Ss. Cyril and Methodius (No. 03-1055/3). All protocols were in accordance with the Declaration of Helsinki, and written informed consent was obtained from each participant in the present study.

### 2.2 Participants

Hypouricemia was defined as serum urate levels equal to or less than 120 μM (approximately 2 mg/dL), according to the clinical practice guideline for renal hypouricemia (Nakayama et al., 2019). Hypouricemia was classified into severe hypouricemia (sU ≤ 60 μM) and moderate hypouricemia (60 < sU ≤ 120 μM); subjects with 120 < sU ≤ 180 μM were considered as mild hypouricemia (Kawamura et al., 2021). As an indicator for increased renal urate excretion—a feature of renal hypouricemia, FE_UA_ ≥ 10% was employed.

In this study, two families were studied. Clinical and biochemical data of the family 1 and 2 are summarized in Table 1 and Supplementary Table S1, respectively. Family 1 originated from Slovakia and the proband 1 exhibited extremely lower serum urate and higher FE_UA_. Family 2 lived in Macedonia and the proband 2 had been a participant in a previous genetic study on nephrolithiasis (Halbritter et al., 2015). At that time, the proband 2 was an 8-year-old boy characterized by mild hypouricemia and found to be a heterozygote for *GLUT9* c.1419+1G>A. For this reason, he and his family members were enrolled in this study in 2021. At our first investigation for the clinical and biochemical re-analyses of the proband 2, he did not meet any criteria of RHUC and had not experience a recurrence of nephrolithiasis; however, in 2022, the proband 2 presented mild renal hypouricemia with nephrolithiasis again.

**TABLE 1:** Pedigree and clinical information of a family with renal hypouricemia.

Family members	RHUC	Sex	Age^a^	Clinical features	sU [μM]^b^	FE_UA_ [%]^b^	sCr [μM]^†^	*GLUT9* genotypes
I:1 (The proband of family 1	Severe	F	51	Congenital convergent strabismus o.sin., hypacusis perceptive l.dx., achalasia of the esophagus, stp.dilatation of the esophagus, art.hypertension, goiter nodosa euf., mild erythrocytosis, discopathy L4/5, without evidence of nephrolithiasis	11 (2017)	>100% (2017)	62 (2017)	c.1419+1A/A (−/−)
					9 (2018)	>100% (2018)	64 (2018)	
					13 (2019)	>100% (2019)	68 (2019)	
					12 (2020)	>100% (2020)	76 (2020)	
					11 (2021)	>100% (2021)	63 (2021)	
II:1 (Daughter of the proband)	Moderate to mild	F	34	Ovarian cysts	85 (2017)	22% (2017)	57 (2017)	NA
				2018: Repeated renal colic, without evidence of nephrolithiasis	135 (2018)	16% (2018)	56 (2018)	
II:2 (Daughter of the proband)	Moderate	F	31	Ovarian cysts	99 (2017)	17% (2017)	65 (2017)	c.1419+1G/A (+/−)
					92 (2018)	16% (2018)	60 (2018)	
					101 (2019)	20% (2019)	67 (2019)	
					103 (2021)	17% (2021)	63 (2021)	
III:1 (Granddaughter of the proband)	Non	F	10	Appropriate physical and mental developement, without evidence of nephrolithiasis	217 (2017)	8% (2017)	42 (2017)	c.1419+1G/G (+/+)
					232 (2021)	8% (2021)	51 (2021)	
III:2 (Granddaughter of the proband)	Non	F	7	Appropriate physical and mental developement, without evidence of nephrolithiasis	267 (2017)	4% (2017)	31 (2017)	c.1419+1G/G (+/+)
					286 (2021)	4% (2021)	54 (2021)	
III:3 (Grandsonr of the proband)	Mild	M	10	Appropriate physical and mental developement, without evidence of nephrolithiasis	142 (2017)	6% (2017)	36 (2017)	c.1419+1G/A (+/−)
					178 (2019)	9% (2019)	45 (2019)	
					147 (2021)	6% (2021)	41 (2021)	
III:4 (Granddaughter of the proband)	Non	F	7	Appropriate physical and mental dvelopement, without evidence of nephrolithiasis	189 (2017)	9% (2017)	27 (2017)	c.1419+1G/A (+/−)
					199 (2019)	7% (2019)	32 (2019)	
					197 (2021)	5% (2021)	34 (2021)	

^a^ Information is as of 2021.

^b^ Year of the measurement was in brackets.

Severe hypouricemia, sU ≤ 60 μM; moderate hypouricemia, 60 < sU ≤ 120 μM; mild hypouricemia, 120 < sU ≤ 180 μM. RHUC, renal hypouricemia; sU, serum urate; FE_UA_, fractional excretion of uric acid; sCr, serum creatinine; NA, not available due to the lack of consent for genetic analyses.

### 2.3 Clinical investigations and genetic analyses

Urate and creatinine concentrations were measured as described previously (Mraz et al., 2015), using a specific enzymatic method and the Jaffé reaction adapted for an auto-analyzer (Hitachi Automatic Analyzer 902; Roche, Basel, Switzerland), respectively. Metabolic investigation for purine metabolism (hypoxanthine and xanthine levels in urine) was also conducted according to the previous study (Mraz et al., 2015). Based on the results of blood and urine tests, FE_UA_ was calculated as follows [urinary urate (μM) ×serum creatinine (μM)]/[serum urate (μM) ×urinary creatinine (μM)] × 100 (%).

Genomic DNA was extracted from a blood sample using a QIAamp DNA Mini Kit (Qiagen GmBH, Hilden, Germany). All coding exons and intron-exon boundaries of *URAT1*/*SLC22A12* and *GLUT9*/*SLC2A9* were amplified from genomic DNA using polymerase chain reaction (PCR) and subsequently purified using a PCR DNA Fragments Extraction Kit (Geneaid, New Taipei City, Taiwan). DNA sequencing was performed on an Applied Biosystems 3130 Genetic Analyzer (Applied Biosystems, Foster City, CA, United States). Primer sequences and PCR conditions used for amplification were described previously (Stiburkova et al., 2011; Stiburkova et al., 2013). The reference genomic sequence was defined as version ENSG00000109667, ENST00000264784 (exons 1–12), and ENST00000506583 (exon 3) for *GLUT9*/*SLC2A9*; ENSG00000197891 and ENST00000377574 for *URAT1*/*SLC22A12*. Then, WES was employed for all the family members of which consent was available. Further details were described in Supplementary Material.

### 2.4 Materials

[8-^14^C]-Uric acid (55 mCi/mmol) was purchased from American Radiolabeled Chemicals (St. Louis, MO, United States). All other chemicals used were commercially available and of analytical grade.

### 2.5 Preparation of GLUT9 variant expression vector

To express human GLUT9 (NM_020041 encoding the transcript variant 1 known as the long form of GLUT9 that is expressed on the basal membrane of renal proximal tubule epithelial cells) fused with EGFP at its C-terminus (GLUT9-EGFP), we used a GLUT9/pEGFP-N1 plasmid that had been constructed in our previous study (Toyoda et al., 2016). The pEGFP-N1 plasmid was used as a control vector. Using a site-directed mutagenesis technique, GLUT9 p.Gly431GlufsTer28 (p.G431fs)/pEGFP-C1 plasmid was generated from the GLUT9 wild-type (WT)/pEGFP-C1 plasmid. In brief, the region of exon 11 (1292G–1419G) was removed from the original plasmid; to unify the linker (GLUT9–EGFP) amino acid sequence between the expression vectors for GLUT9 WT and the p.G431fs variant, unnecessary region [1500T–1620T including the acquired stop codon (1500–1502: TGA)] was also removed. Introduction of each deletion was confirmed by full sequencing using the BigDye Terminator v3.1 (Applied Biosystems) and an Applied Biosystems 3130 Genetic Analyzer (Applied Biosystems) as described previously (Toyoda et al., 2016).

### 2.6 Cell culture and transfection

Human embryonic kidney 293 cell-derived 293A cells were purchased from Life Technologies (Carlsbad, CA, United States) and cultured in Dulbecco’s Modified Eagle’s Medium (DMEM; Nacalai Tesque, Kyoto, Japan) supplemented with 10% fetal bovine serum (Cosmo Bio, Tokyo, Japan), 1% penicillin/streptomycin, 2 mM L-glutamine (Nacalai Tesque), and 1 × Non-Essential Amino Acid (Life Technologies) at 37°C in an atmosphere of 5% CO_2_. Each vector plasmid for GLUT9 WT or p.G431fs was transfected into 293A cells by using polyethyleneimine MAX (PEI-Max; 1 mg/ml in milliQ water, pH 7.0; Polysciences, Warrington, PA, United States) as described previously (Toyoda et al., 2019). The amount of plasmid DNA used for transfection was adjusted among sample groups.

### 2.7 Preparation of whole cell lysates and immunoblotting

Forty-eight hours after the transfection, whole cell lysates were prepared in an ice-cold lysis buffer A containing 50 mM Tris/HCl (pH 7.4), 1 mM dithiothreitol, 1% (w/v) Triton X-100, and a protease inhibitor cocktail for general use (Nacalai Tesque) as described previously (Toyoda et al., 2009). Protein concentration of the whole cell lysate was quantified using a BCA Protein Assay Kit (Pierce, Rockford, IL, United States) with bovine serum albumin as a standard according to the manufacturer’s protocol. For glycosidase treatment, the whole cell lysate samples were incubated with PNGase F (New England Biolabs Japan, Tokyo, Japan) (1.25 U/μg of protein) at 37°C for 10 min as described previously (Toyoda et al., 2019), and then subjected to immunoblotting using a rabbit anti-EGFP polyclonal antibody (A11122; RRID: AB_221569; Life Technologies) to detect GLUT9-EGFP, as described in Supplementary Material.

### 2.8 Confocal laser scanning microscopic observation

For confocal laser scanning microscopy, 48 h after the transfection, 293A cells were fixed with ice-cold methanol for 10 min and then subjected to the visualization of nuclei with TO-PRO-3 Iodide (Molecular Probes, Eugene, OR, United States) as described previously (Toyoda et al., 2019). To analyze the localization of EGFP-fused GLUT9 protein, fluorescence was detected using a FV10i Confocal Laser Scanning Microscope (Olympus, Tokyo, Japan).

### 2.9 Urate transport assay

The [8-^14^C]-urate transport assay of GLUT9 was conducted using GLUT9-expressing 293A cells according to our previous studies (Miyata et al., 2016; Toyoda et al., 2020), with some minor modifications. In brief, 48 h after plasmid transfection, the cells were washed twice with high-potassium transport buffer (Buffer K-high: 145.4 mM KCl, .8 mM MgSO_4_, 1.8 mM CaCl_2_, 25 mM HEPES, 25 mM Tris, 5 mM D-glucose, and pH 7.4) and pre-incubated in Buffer K-high for 10 min at 37°C. The buffer was then exchanged with pre-warmed fresh Buffer K-high containing 10 μM [8-^14^C]-urate. The cells were further incubated for 5 min. The cells were subsequently washed three times with ice-cold Buffer K-high and then lysed with 500 μl of .2 M NaOH on ice with gentle shaking for 1 h. The resulting lysates were neutralized with 100 μl of 1 M HCl. We then measured the radioactivity in the lysate using a liquid scintillator (Tri-Carb 3110TR; PerkinElmer, Waltham, MA, United States). The protein concentrations were determined using the Pierce™ BCA Protein Assay Kit. The urate transport activity was calculated as the incorporation rate (pmol/min/mg protein).

### 2.10 Statistical analysis

All statistical analyses were performed by using EXCEL 2019 (Microsoft, Redmond, WA, United States) with Statcel4 add-in software (OMS publishing, Saitama, Japan). GraphPad Prism 8 (GraphPad Software, San Diego, CA, United States) was used for graph making. The numbers of biological replicates (*n*) were described in the figure legends. When analyzing multiple groups, the similarity of variance between groups was compared using Bartlett’s test. Based on the result, a non-parametric Steel test was used in this study. Statistical significance was defined in terms of *p* values less than .05 or .01.

## 3 Results

### 3.1 Subjects with familial renal hypouricemia

Clinical information including serum urate levels of the Slovak family (termed family 1) are summarized in Table 1. Repeated biochemical analysis of the proband 1 in the family 1 (a 51-year-old Slovakian woman with more than a decade history of severe hypouricemia, which was found at least when she was 42-year-old) showed extremely low serum urate (9–13 μM; .15–.22 mg/dL) with an increased FE_UA_ (>100%). These shifts in serum urate and FE_UA_ were consistent with typical patterns observed in RHUC, especially type 2 rather than type 1 (Kawamura et al., 2021). In addition to the hypouricemia, the proband 1 also suffered from congenital disorders including convergent strabismus of the left eye, congenital hypoacusis of right ear, and esophageal achalasia; however, her values of estimated glomerular filtration rate (eGFR, 114 ml/min/1.73 m^2^) were in normal range with neither reported history of nephrolithiasis nor acute renal injury, indicating that her kidneys must have functioned normally except for renal urate handling.

**FIGURE 1:**
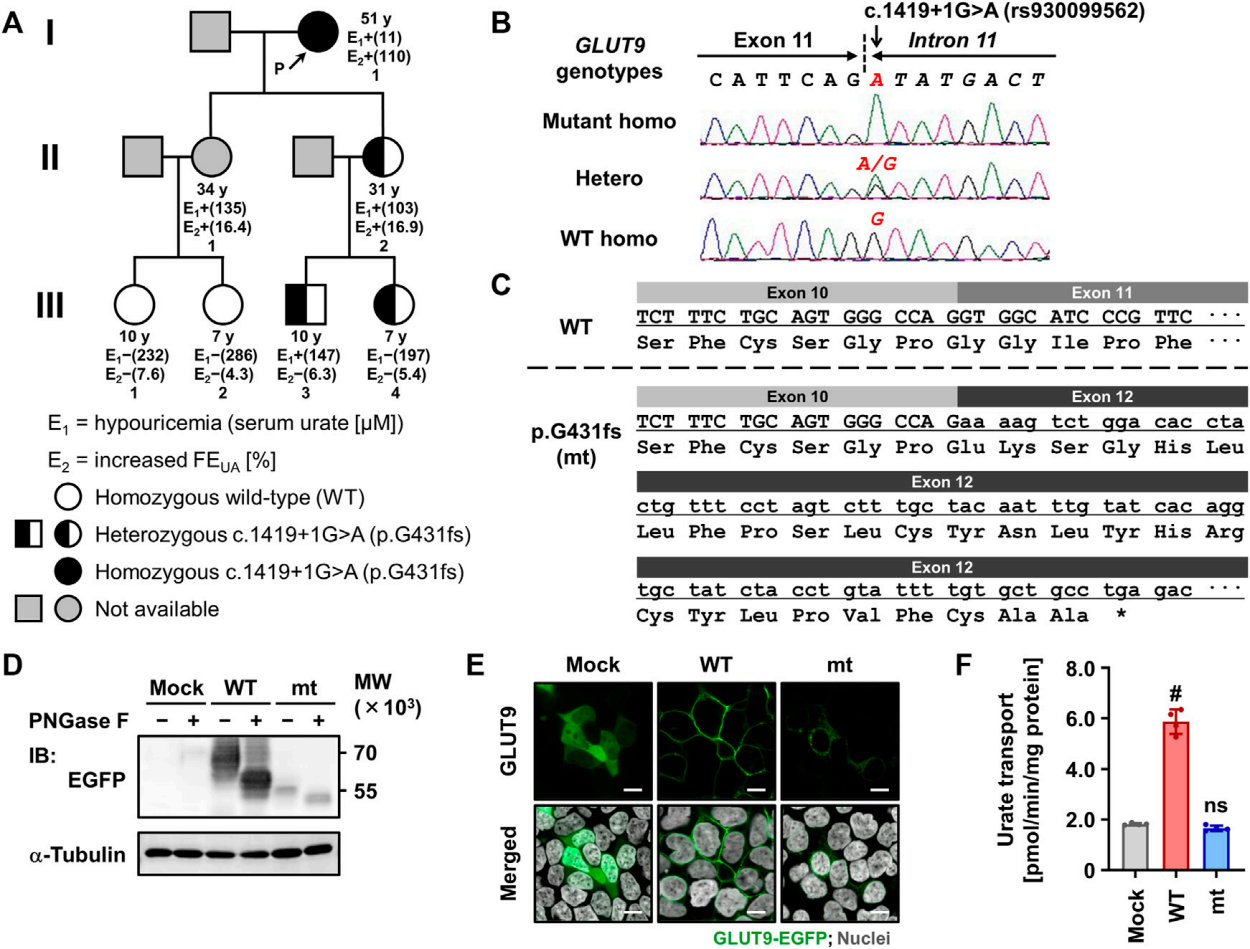
Identification and functional characterization of the exon-skipping dysfunctional variant c.1419+1G>A (p.G431fs) in *GLUT9*/*SLC2A9*. **(A)** Pedigree of a family with renal hypouricemia. Representative values of serum urate and fractional excretion of uric acid (FE_UA_) for each subject, which were obtained in 2021 (except for II:1 whose data were obtained in 2018), are shown. Details of clinical information are summarized in Table 1. **(B)** Representative electropherograms of partial sequences of *GLUT9*/*SLC2A9* showing the homozygotes/heterozygous point mutations discovered in our present study. **(C)** Impact of the c.1419+1G>A variant on the amino-acid sequence of GLUT9 protein showing the exon 11 skipping, frame-shift, and truncation due to a termination codon (*). **(D)** Immunoblot of whole cell lysate samples showing the lower protein levels and incomplete maturation of the p.G431fs variant compared with GLUT9 WT. 48 h after the transfection, EGFP-tagged GLUT9 proteins expressed in 293A cells were detected using an anti-EGFP antibody. *α*-Tubulin, a loading control. WT, wild-type; mt, p.G431fs variant. **(E)** Confocal microscopy for intracellular localization showing the deficiency in plasma membrane localization of the p.G431fs variant contrary to WT. Nuclei were stained with TO-PRO-3 iodide (gray). Bars, 10 μm. **(F)** Urate transport activities. Data are expressed as the mean ± SD. *n* = 4. ^#^, *p* < .05; ns, not significantly different between groups (steel test; vs. mock).

The pedigree of this family 1 is presented in Figure 1A. Both of the proband’s daughters (aged 34- and 31-years-old) exhibited mild hypouricemia and hyperuricosuria (sU, 135 and 103 μM; FE_UA_, 16% and 17%, respectively); none of the grandchildren of the proband presented hypouricemia. Further metabolomic investigations for purine metabolism of each subject in this family found normal urinary excretion of xanthine and hypoxanthine, indicating that hypouricemia in the proband and her family relatives would not been due to the decreasing of uric acid production. Based on these pieces of information, we concluded that the hypouricemia found in the family 1 was RHUC. To investigate the genetic cause of this inheritable RHUC, we focused on renal urate handling and its regulatory machineries as described below.

### 3.2 Identification of *GLUT9* c.1419+1G>A (p.G431fs) as a disease-causative variation

Since *URAT1* (located on the human chromosome 11) and *GLUT9* (located on the human chromosome 4) are known as causative genes for RHUC type 1 and type 2, respectively, we examined genotypes of these two genes in the family 1 to explore the possible genetic cause(s) of the familial RHUC. First, we conducted a series of target sequencing for all the coding regions of *URAT1* (10 exons) and *GLUT9* (12 exons). As a result, no possible candidate was found in *URAT1*; however, in *GLUT9*, a putative causal variation—c.1419+1G>A (rs930099562)—was identified in an exon–intron boundary region (Figures 1A, B). Indeed, proband 1 in the family 1 was homozygous for this genetic variation (c.1419+1A/A) and she had no non-synonymous *GLUT9* variants that are reportedly related with RHUC. Regarding her two daughters with RHUC, although genetic information was not available in the elder sister, the younger sister was heterozygous for the locus (c.1419+1G/A), which was consistent with the expected relationship between the genotype and phenotype. Regarding the four grandchildren, no RHUC was found; however, subjects harboring one c.1419+1A allele (III:3 and III:4) exhibited lower serum urate levels (mild hypouricemia and its borderline, respectively) compared with subjects without the mutant allele (III:1 and III:2). Additionally, WES analyses for the family 1 relatives revealed that the genetic variation c.1419+1G>A in *GLUT9* was the top candidate for the causality.

To validate the causal relationship between *GLUT9* c.1419+1G>A and RHUC in a clinico-genetic manner, we addressed other subjects. Literature search revealed that in a previous study (Halbritter et al., 2015), *GLUT9* c.1419+1G>A was found in a Macedonian individual (an 8-year-old boy at that time) who was diagnosed with idiopathic hypouricemia and nephrolithiasis. In 2012, he (proband 2) exhibited mild hypouricemia (sU, 123 μM; FE_UA_, 19.8%). While his serum urate levels were sometimes in normal range, mild hypouricemia phenotypes (sU, 133 μM; FE_UA_, 22.3%) were observed in a recent measurement that we herein conducted in 2022. Our genetic analyses confirmed that he was heterozygous for the locus (c.1419+1G/A). With his family relatives, only his father has the c.1419+1A allele as a heterozygote and exhibited moderate hypouricemia phenotypes (sU, 111 μM; FE_UA_, 15.5%). Detailed information on this Macedonian family (family 2) are summarized in Supplementary Table S1. In addition, there was no evidence of genetic relatedness (i.e., kinship coefficient less than 0) between the family 1 (from Slovakia) and the family 2 (from Macedonia) following analysis with PLINK 2.00 alpha using WES data (Supplementary Table S2).

Given that c.1419+1G>A disrupts the canonical 5′ splice site localized between exon 11 and intron 11 in *GLUT9* (Figure 1B), this mutation would affect the splicing of GLUT9 mRNA. Based on a theory (Anna and Monika, 2018), this kind of mutation would expectedly result in the entire skipping of a neighboring exon from the transcript after the maturation of pre-mRNA (Figure 1C). In other words, c.1419+1G>A could lead to the exon 11 skipping resulting in a frameshift and premature termination (p.Gly431GlufsTer28, p.G431fs).

### 3.3 Identification of GLUT9 p.G431fs as a functionally-null variant

**FIGURE 2:**
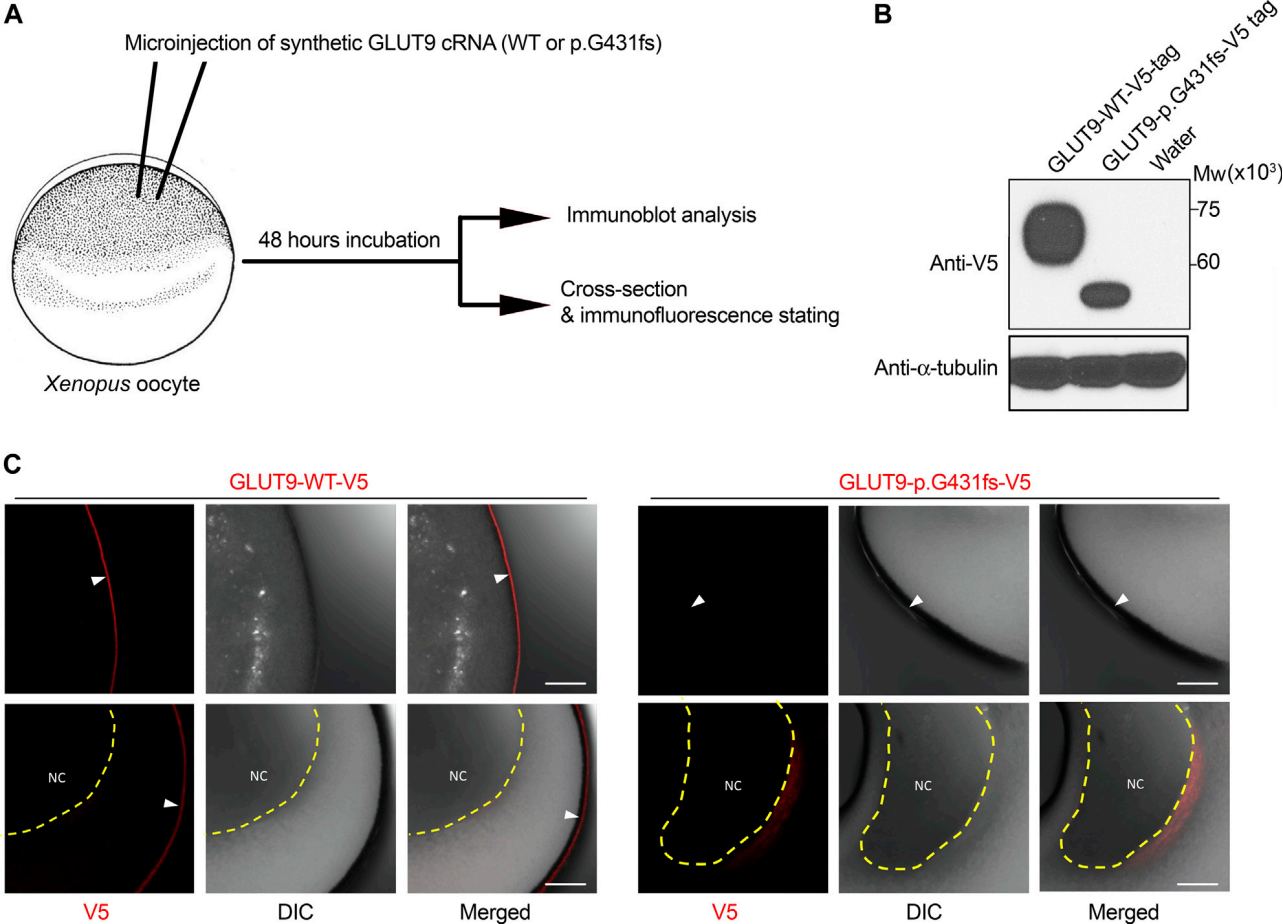
Expression analysis of GLUT9 wild type and p.G431fs mutant in *Xenopus* oocytes. **(A)** Schematic illustration of experimental procedures. In this study, 5 ng cRNA of GLUT9 wild-type (WT) or p.G431fs was injected into oocytes. After 48 h incubation, the oocytes were subjected to immunoblot analysis and immunofluorescence stating. Details are described in Supplementary Material. **(B)** Immunoblot detection of GLUT9 protein transiently expressed in oocytes using an anti-V5 antibody. *α*-Tubulin, a loading control. **(C)** Immunofluorescence stating of GLUT9 protein transiently expressed in oocytes. GLUT9 WT was localized on the plasma membrane of oocytes; however, GLUT9 p.G431fs hardly reached the plasma membrane. White arrow heads indicate plasma membrane; yellow dashed lines surround nucleus (NC). DIC, differential interference contrast. Bars, 10 μm.

To investigate the effect of the novel exon-skipping variation (p.G431fs) on the intracellular processing and function of GLUT9 protein, we performed a series of biochemical analyses using transiently GLUT9-expressing mammalian 293A cells (Figures 1D–F). First, immunoblotting for *N*-glycosidase (PNGase F)-treated whole cell lysates (Figure 1D) demonstrated that the p.G431fs variant had lower levels of GLUT9 and did not matured as an *N*-linked glycosylated protein as evidenced by its truncated forms with weaker band intensity compared with that of GLUT9 WT. Next, confocal microscopy (Figure 1E) showed that unlike the case of GLUT9 WT, the p.G431fs variant showed greatly reduced localization on the plasma membrane of the cells. Such insufficient intracellular processing of the p.G431fs variant was also observed in another expression system using *Xenopus* oocytes (Figure 2). Furthermore, we conducted a mammalian cell-based functional assay in a high-potassium buffer condition that depolarizes plasma membrane, which enabled us to evaluate GLUT9 function as [8-^14^C]-urate uptake into cells (Nakayama et al., 2017). The functional assay revealed that, contrary to GLUT9 WT-expressing cells, urate transport activities in the p.G431fs variant-expressing cells were not significantly different from those in control cells, indicating that the p.G431fs variant was functionally null as a urate transporter (Figure 1F). As the cellular function of transporter protein is affected by both its quantity (cellular protein level) and quality (transport activity per molecule), this result, reflecting the effects of the variant on both of the protein amount and activity, was consistent with the results of biochemical analyses (Figures 1D, E). We, therefore, concluded that this GLUT9 mutation is the cause of familial renal hypouricemia in this study.

**FIGURE 3:**
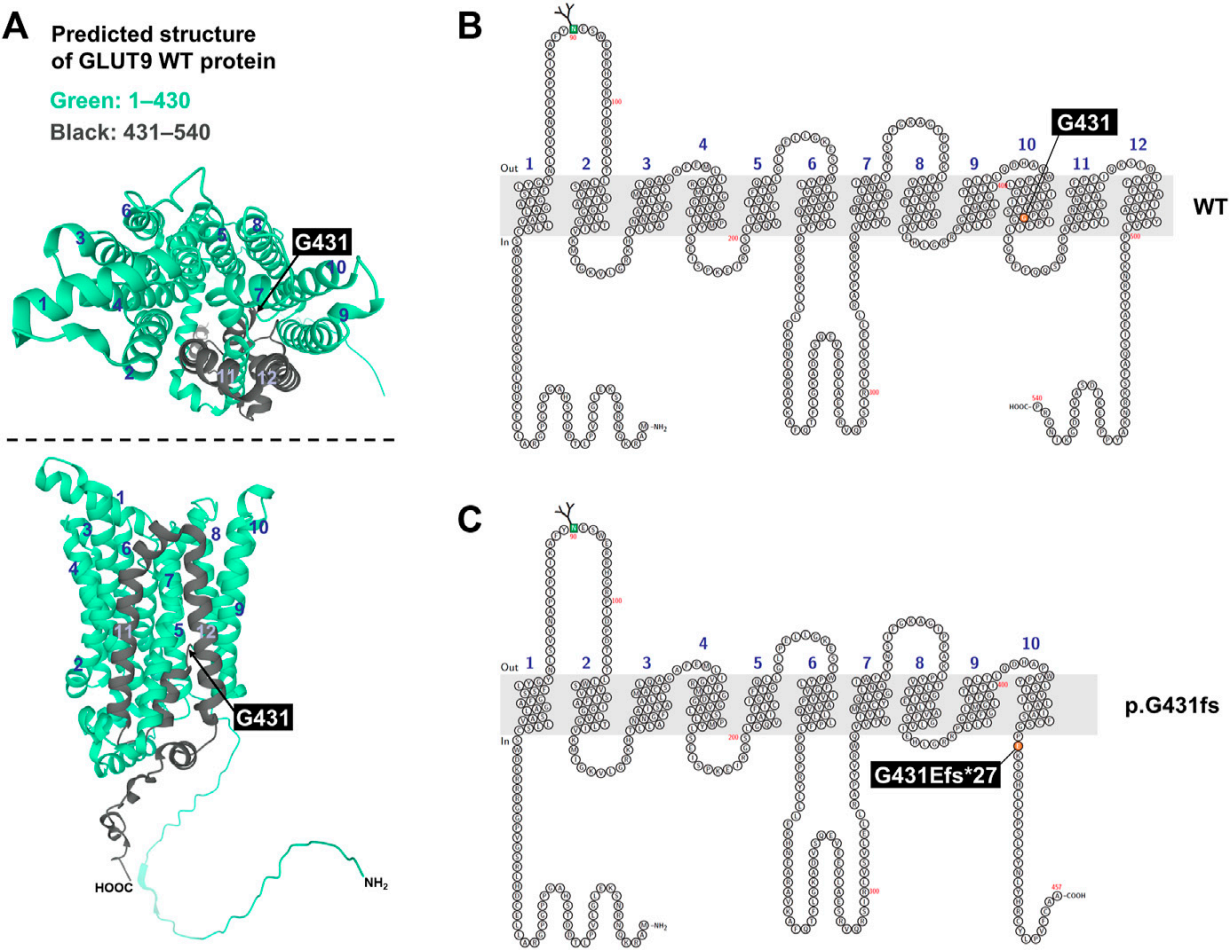
Prediction of human GLUT9 protein structure. **(A)** Predicted structure of GLUT9. Data are from AlphaFold Protein Structure Database (https://alphafold.ebi.ac.uk/; accessed on 22 March 2022). Original amino acids M1–P430 and G431–P540 in GLUT9 wild-type (WT) protein were colored green and black, respectively. Upper panel, top view; lower panel, side view. **(B,C)** Schematic illustration of predicted topology of GLUT9 WT **(B)** and p.G431fs (exon skipping variant) **(C)**. Each membrane topology was predicted by Protter (http://wlab.ethz.ch/protter/start/; accessed on 22 March 2022). Light gray area means plasma membrane. Numbers indicates each transmembrane domain.

Additionally, given a predicted structure of GLUT9 protein by AlphaFold (Figure 3A), G431 is located in the lower part of the transmembrane domain (TMD) 10 and the exon skipping variant p.G431fs causes a frameshift that replaces original amino acids (431G–540P, colored black in (Figure 3A) with a different shorter amino-acid chain, resulting in the complete loss of TMD11, TMD12, and the original intracellular domain at C-terminal. This structural change was also supported by another structural prediction by Protter based on amino acid sequence and its hydrophobicity (Figures 3B, C). Due to the lack of such domains, the GLUT9 variant would be recognized as a misfolded protein by cellular quality control systems, which result in the loss of cellular function.

## 4 Discussion

In this study, *via* clinico-genetic approaches, we investigated the latent causative factor of RHUC in the family from Slovakia of which proband exhibited severe RHUC phenotypes (Table 1). As a result, we successfully identified the causal relationship with *GLUT9* c.1419+1G>A (Figures 1A, B). Besides, this causality was supported by another Macedonian family characterized by moderate or mild RHUC (Supplementary Table S1). Further analyses revealed that this intronic variant could encode a splicing variant with a premature terminate codon caused by exon 11 skipping—p.Gly431GlufsTer28 (Figure 1C). Biochemical and functional assays demonstrated that this GLUT9 variant was functionally null (Figures 1D–F). Given that no possible causative variations were found in *URAT1* in the participants of this study, our results indicated that all the cases of RHUC studied in this study were type 2.

Our findings extend the understanding on the importance of mRNA splicing in disease pathogenesis (Anna and Monika, 2018; Park et al., 2018). The G-to-A intronic substitution (rs930099562) within the 5′ splice site in an exon–intron boundary region in *GLUT9* could lead to the production of an aberrant splicing variant, containing a premature termination codon, that is functionally null (Figure 1). Although this *GLUT9* variant is extremely rare (*i.e.*, minor allele frequency was .000004–.000007 according to the NCBI dbSNP), considering a recent study reporting that a functionally-null intronic variant (c.506+1G>A) in *URAT1* is a causative factor for RHUA type 1 (Kawamura et al., 2020), our discovery supports the notion that genetic variations in the intronic (non-cording) regions of *GLUT9* as well as *URAT1* should also be kept in mind during diagnostic procedures for renal hypouricemia.

Clinical data obtained in this study strongly support the notion that GLUT9 could have a greater effect to net renal urate re-uptake into blood compared with URAT1. Our data showed that the complete loss-of-function of GLUT9 in humans could result in extremely low serum urate and extremely high FE_UA_ as observed in the proband 1 (sU, 9–13 μM; FE_UA_ >100%). A similar severe phenotype (sU, approximately 6 μM; FE_UA_, >150%) was previously reported in a RHUC patient who was homozygous for the 36-kb deletion in *GLUT9* (Dinour et al., 2010); this deletion provided the exon 7 skipping variant (p.Ala272AspfsTer42) that would be functionally null. Moreover, two siblings who were homozygous for GLUT9 p.Ile118HisfsTer27 reportedly exhibited severe hypouricemia (sU, around 10 μM) and hyperuricosuria (FE_UA_, >100%) (Stiburkova et al., 2011). On the other hand, given typical clinical cases for RHUC type 1 (Ichida et al., 2004; Cheong et al., 2005; Wakida et al., 2005; Kawamura et al., 2021), the patients with complete loss-of-function of URAT1 seem to have somewhat higher serum urate and lower FE_UA_ as compared with RHUC type 2; this tendency, especially regarding FE_UA_, is also noted by our previous discussion (Kawamura et al., 2021). A plausible explanation for this point would be that URAT1 but not GLUT9 might have backup machinery. Indeed, recent studies revealed that like URAT1, organic anion transporter 10 (also known as SLC22A13) has a physiological role as a renal urate re-absorber expressed on the apical side of renal proximal tubular cells though its contribution on renal urate handling is smaller than that of URAT1 (Higashino et al., 2020; Toyoda et al., 2022). By contrast, on the basal side of the cells, no physiologically-important urate transporters have been identified to date, except for GLUT9.

The clinical phenotype severity of the familial RHUC type 2 found in this study had a good correlation with the number of the mutation alleles at *GLUT9* c.1419+1G>A (Table 1; Supplementary Table S1), although sample size was small. Indeed, the proband 1 (I:1) in the family 1, who was homozygous for this mutant and thereby lost her GLUT9 function, exhibited severe phenotypes. With the elder daughter of the proband 1 (II:1 in the family 1), no genetic information was examined; however, their blood relationship suggested that the older daughter was at least heterozygous for this allele, which was consistent with her RHUC phenotypes (moderate to mild). This association was also the case with the other heterozygous subjects; there was one exception—III:4 in the family 1, but serum urate (189–199 μM) and FE_UA_ (5%–9%) of this subject were borderline levels. On the other hand, whether age and sex could have influenced the phenotype severity remains unclear. The potential effects of such non-genetic factors on RHUC phenotypes will be a future topic.

In conclusion, we herein described a typical case of familial RHUC type 2 associated with the complete dysfunction of GLUT9. This study revealed that *GLUT9* c.1419+1G>A is a functionally-null and causative variation for RHUC type 2. In this context, not only exonic regions but also non-coding regions of *GLUT9* as well as *URAT1* should be addressed in genetic diagnosis for hypouricemia. Our findings will extend the understanding of pathophysiological importance of genetic variations in *GLUT9* as well as aetiology of RHUC.

## Data Availability

The datasets for this article are not publicly available due to concerns regarding participant/patient anonymity. Requests to access the datasets should be directed to the corresponding author.
