# Supplementary material for: Identification of a dysfunctional exon-skipping splice variant in GLUT9/SLC2A9 causal for renal hypouricemia type 2
Source: Front Genet. 2023 Jan 17;13:1048330. doi: 10.3389/fgene.2022.1048330 (PMC9887137; doi:10.3389/fgene.2022.1048330)
Supplement: Supplementary file 1 [file DataSheet1.PDF]

Supplementary Material

1 Supplementary Methods

1.1 Whole Exome Sequencing

Genomic DNA was obtained from peripheral blood leukocytes of each subject. Quality and quantity of extracted DNA were assessed by an OD260/280 ratio of 1.8–2.0, 1% agarose gel electrophoresis, and PicoGreen® dsDNA Assay (Invitrogen, Waltham, MA, USA). Sequencing libraries were generated using the SureSelect All Exon V5 kit (Agilent Technologies, Santa Clara, CA, USA), followed by sequencing with the NovaSeq 6000 S4 (Illumina, San Diego, CA, USA). Image analysis and base calling were performed with the pipeline software under default parameters. Mapping was completed using the human reference genome assembly (GRCh37/hg19); all variants were called and annotated using the Collaborative Bioinformatics Resource (CCBR) Pipeliner (https://github.com/CCBR/Pipeliner). The overall variant-identifying process referred to the standard guidelines of investigating variants for Mendelian disorders from WES data (Yang et al., 2013; MacArthur et al., 2014). We performed the analysis, assuming an autosomal recessive or X-linked recessive pattern, according to the observed inheritance mode in hereditary RHUC (Sperling, 2006). *GLUT9* variants identified in this study were annotated using Annotation, VisUAlization, and Impact Analysis webserver (AVIA v.2.0) (Vuong et al., 2015). Information on minor allele frequency was collected from the NCBI dbSNP (https://www.ncbi.nlm.nih.gov/snp/rs930099562; accessed June, 2022). The genetic relatedness was calculated by PLINK 2.0 and assessed by Identity-by-descent.

1.2 Immunoblotting for GLUT9-expressing 293A cells

Expression of GLUT9 protein in whole cell lysates of GLUT9-expressing 293A cells was examined by immunoblotting as described previously (Toyoda et al., 2019), with minor modifications. In brief, the prepared samples were mixed with a sodium dodecyl sulfate polyacrylamide gel electrophoresis sample buffer solution containing 10% 2-mercaptoethanol, separated by electrophoresis on poly-acrylamide gels, and then transferred to polyvinylidene difluoride membranes (Immobilon; Millipore, Billerica, MA, USA) by electroblotting at 15 V for 60 min. For blocking, the membrane was incubated in Tris-buffered saline containing 0.05% Tween 20 and 3% bovine serum albumin (Nacalai Tesque, Kyoto, Japan) (TBST-3% BSA). After overnight incubation at room temperature, blots were probed with a rabbit anti-EGFP polyclonal antibody (A11122; RRID: AB_221569; Life Technologies, Carlsbad, CA, USA; diluted 1,500-fold in TBST-0.1% BSA), or a rabbit anti-α-tubulin antibody (ab15246; RRID: AB_301787; Abcam, Cambridge, MA, USA; diluted 1,000-fold) followed by incubation with a donkey anti-rabbit immunoglobulin G (IgG)-horseradish peroxidase (HRP)-conjugated antibody (NA934V; RRID: AB_772206; diluted 4,000-fold for GLUT9-EGFP or 3,000-fold for α-tubulin). HRP-dependent luminescence was developed using the ECL™ Prime Western Blotting Detection Reagent (GE Healthcare UK, Buckinghamshire, UK) and detected using a multi-imaging Analyzer Fusion Solo 4™ system (Vilber Lourmat, Eberhardzell, Germany).

1.3 Generation and *in vitro* transcription of GLUT9 expression vectors for *Xenopus* oocytes

The recombinant plasmid GLUT9 wild-type (WT)-EGFP/pcDNA-DEST47 (RRID: Addgene_18730) (Takanaga et al., 2008) was kindly gifted from Dr. Wolf Frommer. GLUT9 open reading frame was

amplified from this template vector with V5 tag sequence; then the amplicon was inserted into a pCS107 vector using restriction enzyme sites of *Cla* I and *Not* I. From the resulting GLUT9 WT-V5/pCS107 vector, GLUT9 p.G431fs-V5/pCS107 vector was generated using a site-directed mutagenesis technique, followed by the confirmation of sequence by Sanger sequencing. All the plasmids used in the following experiments were purified with a GenElute Endotoxin-free Plasmid Maxiprep Kit (NA0310; Sigma-Aldrich, Burlington, MA, USA). To synthesize cRNA of GLUT9, each plasmid construct was linearized by a restriction enzyme, *Asp*718 I (Roche, Indianapolis, IN, USA), and then transcribed *in vitro* with the SP6 mMESSAGE mMACHINE Kit (#AM1340; Ambion, Austin, TX, USA) according to the manufacture's instruction.

1.4 Injection of GLUT9 cRNA into *Xenopus* oocytes

Oocytes were prepared from anesthetized *Xenopus laevis* (Nasco, Fort Atkinson, WI, USA) and defoliculated as described previously (Sive et al., 2000). Stages V–VI oocytes were injected with 5 ng cRNA of GLUT9 WT or p.G431fs in 5 nL of RNA free water, or equivalent volume of water for control, followed by incubation at 20°C for 48 h in an oocyte culture medium [50% Liebovitz L-15 medium, 15 mM HEPES, 1 mg/ml insulin stock, 50 U/mL Nystatin (N1638; Sigma-Aldrich, Burlington, MA, USA), 100 U/mL Penicillin/streptomycin (15070063; Life Technologies), and 0.1 mg/mL gentamycin].

Animal care and use for this study was performed in accordance with the recommendations of AAALAC for the care and use of laboratory animals in an AAALAC approved facility. Experimental procedures were specifically approved by the animal care & use committee of the of the National Cancer Institute-Frederick (ASP #18-433) in compliance with AAALAC guidelines.

1.5 Confocal microscopy for GLUT9-expressing oocytes

Immunofluorescence staining and detection were conducted as described previously (Yoon et al., 2021), with minor modifications. Briefly, GLUT9-expressing *Xenopus* oocytes were fixed with 4% paraformaldehyde in PBS at 4°C overnight. Then, the oocytes were embedded in 4% low melting agarose gel and were sectioned with a thickness of 100-µm with a LEICA VT 1200S vibratome (Leica, Bensheim, Germany). The sections were treated with an anti-V5 tag antibody (mouse monoclonal) (G189; Applied Biological Materials, Richmond, Canada; diluted 500-fold) and a donkey anti-mouse IgG (H+L) highly cross-adsorbed secondary antibody, Alexa Fluor™ plus 594 (A32744; RRID: AB_2762826; Invitrogen, Waltham, MA, USA; diluted 500-fold) at 4°C overnight. After washing with PBS containing 0.1% Triton-X100, the sections were mounted in Fluoro-Gel (Electron Microscopy Sciences, Hatfield, PA, USA) and imaged using a Zeiss LSM-880 laser scanning confocal microscope (Carl Zeiss, Jena, Germany).

1.6 Lysate preparation and immunoblotting for GLUT9-expressing oocytes

From ten oocytes for each experimental group, lysates were prepared in an ice-cold TNSG buffer [20 mM Tris/HCl (pH 7.5), 137 mM NaCl, and 1% NP-40], as described previously (Yoon et al., 2018). According to a previous study (Yoon et al., 2021), with minor modifications, the lysates were separated by electrophoresis on a 10% SDS poly-acrylamide gel, and then transferred onto polyvinylidene difluoride membranes (GE Healthcare Life Science, Pittsburgh, PA, USA) by electroblotting at 100 V for 180 min. After blocking with 10% skim milk in TBST, blots were probed with an anti-V5-

horseradish peroxidase (HRP)-conjugated antibody (V5 Tag Monoclonal Antibody, HRP; R961-25; RRID: AB_2556565; Invitrogen; diluted 2000-fold) overnight. Then, HRP-dependent luminescence was developed using ECL Western Blotting Substrate (Pierce Biotechnology, Rockford, IL, USA) and detected using an ImageQuant™ LAS 4000 (GE Healthcare Life Science, Pittsburgh, PA, USA).

1.7 Schematic illustration of GLUT9 protein

A predicted 3D structure of GLUT9 protein was obtained from AlphaFold Protein Structure Database (https://alphafold.ebi.ac.uk/) (Jumper et al., 2021; Varadi et al., 2022) and processed using the related online application. Also, using the open-source tool Protter (Omasits et al., 2014), 2D topologies of GLUT9 WT and p.G431fs variant were predicted based on amino acid sequences, and the obtained-topology data were plotted and modified using the T(E)Xtopo package (Beitz, 2000).

2 Supplementary Tables

Supplementary Table S1. Pedigree and clinical information of a Macedonian family with renal hypouricemia.

Family members	RHUC	Sex	Age [#]	Clinical features	sU [μM] [†]	FE_{UA} [%] [†]	sCr [μM] [†]	*GLUT9* genotypes
I:1<br>(Father of the proband)	Moderate	M	41	Normal U/S<br>Without nephrolithiasis	111 (2022)	16% (2022)	68	c.1419+1G/A<br>(+/−)
I:2<br>(Mother of the proband)	Non	F	43	Normal U/S	217 (2022)	7% (2022)	61	c.1419+1G/G<br>(+/+)
II:1<br>(The proband of family 2)	Mild	M	18	Nephrolithiasis lat eral	123 (2012)<br>266 (2020)<br>133 (2022)	20% (2012)<br>4% (2020)<br>22% (2022)	51	c.1419+1G/A<br>(+/−)
II:2<br>(Sibling of the proband)	Non	F	12	Normal U/S	300 (2022)	5% (2022)	44	c.1419+1G/G<br>(+/+)

[#], Information is as of 2022; [†], year of the measurement was in brackets. Severe hypouricemia, sU ≤ 60 μM; moderate hypouricemia, 60 < sU ≤ 120 μM; mild hypouricemia, 120 < sU ≤ 180 μM. RHUC, renal hypouricemia; BYR, year of birth; sU, serum urate; FE_{UA}, fractional excretion of uric acid; sCr, serum creatinine; U/S, ultrasound.

Supplementary Table S2. Calculated relatedness.

Subjects	Subjects (reference)	NSNP	Kinship coefficient	Relationship
III:3 (Family 1)	III:4 (Family 1)	196,810	0.242283	Full siblings
II:2 (Family 2)	II:1 (Family 2)	196,810	0.226967	Full siblings
I:2 (Family 2)	II:2 (Family 2)	196,810	0.225247	Parent-child
I:2 (Family 2)	II:1 (Family 2)	196,810	0.222705	Parent-child
I:1 (Family 2)	II:2 (Family 2)	196,810	0.220377	Parent-child
I:1 (Family 2)	II:1 (Family 2)	196,810	0.219279	Parent-child
III:2 (Family 1)	III:1 (Family 1)	196,810	0.218489	Full siblings
II:2 (Family 1)	I:1 (Family 1)	196,810	0.201383	Parent-child
III:2 (Family 1)	II:2 (Family 1)	196,810	0.199099	Parent-child
III:1 (Family 1)	II:2 (Family 1)	196,810	0.197858	Parent-child
II:2 (Family 1)	III:3 (Family 1)	196,810	0.107805	Aunt-Nephew
I:1 (Family 1)	III:3 (Family 1)	196,810	0.104837	Grandparent-Grandchild
I:1 (Family 1)	III:4 (Family 1)	196,810	0.083519	Grandparent-Grandchild
II:2 (Family 1)	III:4 (Family 1)	196,810	0.078317	Aunt-Nephew
III:2 (Family 1)	I:1 (Family 1)	196,810	0.070967	Grandparent-Grandchild
III:1 (Family 1)	I:1 (Family 1)	196,810	0.069918	Grandparent-Grandchild
III:1 (Family 1)	III:3 (Family 1)	196,810	0.057009	First-Cousin
III:2 (Family 1)	III:3 (Family 1)	196,810	0.054089	First-Cousin
III:1 (Family 1)	III:4 (Family 1)	196,810	0.050328	First-Cousin
III:2 (Family 1)	III:4 (Family 1)	196,810	0.049985	First-Cousin
I:1 (Family 2)	I:2 (Family 2)	196,810	0.005690	Unrelated
III:1 (Family 1)	II:2 (Family 2)	196,810	−0.034458	Unrelated
III:1 (Family 1)	I:2 (Family 2)	196,810	−0.035933	Unrelated
III:3 (Family 1)	II:2 (Family 2)	196,810	−0.037651	Unrelated
III:3 (Family 1)	I:2 (Family 2)	196,810	−0.037692	Unrelated
I:1 (Family 2)	III:3 (Family 1)	196,810	−0.040486	Unrelated
III:4 (Family 1)	II:2 (Family 2)	196,810	−0.041110	Unrelated
I:1 (Family 2)	III:1 (Family 1)	196,810	−0.042284	Unrelated
III:3 (Family 1)	II:1 (Family 2)	196,810	−0.044057	Unrelated
III:4 (Family 1)	I:2 (Family 2)	196,810	−0.045159	Unrelated
III:1 (Family 1)	II:1 (Family 2)	196,810	−0.046642	Unrelated
I:1 (Family 2)	III:4 (Family 1)	196,810	−0.050402	Unrelated
III:2 (Family 1)	I:2 (Family 2)	196,810	−0.051372	Unrelated
III:4 (Family 1)	II:1 (Family 2)	196,810	−0.051776	Unrelated
I:1 (Family 2)	III:2 (Family 1)	196,810	−0.052039	Unrelated
I:1 (Family 2)	III:2 (Family 1)	196,810	−0.052039	Unrelated
III:2 (Family 1)	II:2 (Family 2)	196,810	−0.054024	Unrelated
III:2 (Family 1)	II:1 (Family 2)	196,810	−0.058674	Unrelated
I:1 (Family 1)	II:2 (Family 2)	196,810	−0.070430	Unrelated
II:2 (Family 1)	II:2 (Family 2)	196,810	−0.071009	Unrelated
II:2 (Family 1)	I:2 (Family 2)	196,810	−0.071051	Unrelated
I:1 (Family 1)	I:2 (Family 2)	196,810	−0.074456	Unrelated
I:1 (Family 2)	II:2 (Family 1)	196,810	−0.075034	Unrelated
I:1 (Family 2)	I:1 (Family 1)	196,810	−0.075731	Unrelated
II:2 (Family 1)	II:1 (Family 2)	196,810	−0.076909	Unrelated
I:1 (Family 1)	II:1 (Family 2)	196,810	−0.078579	Unrelated

NSNP, number of single nucleotide polymorphisms used for the analysis.

3 Supplementary References

Beitz, E. (2000). T(E)Xtopo: shaded membrane protein topology plots in LAT(E)X2epsilon. *Bioinformatics* 16(11)**,** 1050-1051.

Jumper, J., Evans, R., Pritzel, A., Green, T., Figurnov, M., Ronneberger, O., et al. (2021). Highly accurate protein structure prediction with AlphaFold. *Nature* 596(7873)**,** 583-589. doi: 10.1038/s41586-021-03819-2.

MacArthur, D.G., Manolio, T.A., Dimmock, D.P., Rehm, H.L., Shendure, J., Abecasis, G.R., et al. (2014). Guidelines for investigating causality of sequence variants in human disease. *Nature* 508(7497)**,** 469-476. doi: 10.1038/nature13127.

Omasits, U., Ahrens, C.H., Muller, S., and Wollscheid, B. (2014). Protter: interactive protein feature visualization and integration with experimental proteomic data. *Bioinformatics* 30(6)**,** 884-886. doi: 10.1093/bioinformatics/btt607.

Sive, H.L., Grainger, R.M., and Harland, R.M. (2000). *Early Development of Xenopus Laevis: A Laboratory Manual.* Cold Spring Harbor, NY: Cold Spring Harbor Laboratory Press.

Sperling, O. (2006). Hereditary renal hypouricemia. *Mol Genet Metab* 89(1-2)**,** 14-18. doi: 10.1016/j.ymgme.2006.03.015.

Takanaga, H., Chaudhuri, B., and Frommer, W.B. (2008). GLUT1 and GLUT9 as major contributors to glucose influx in HepG2 cells identified by a high sensitivity intramolecular FRET glucose sensor. *Biochim Biophys Acta* 1778(4)**,** 1091-1099. doi: 10.1016/j.bbamem.2007.11.015.

Toyoda, Y., Mancikova, A., Krylov, V., Morimoto, K., Pavelcova, K., Bohata, J., et al. (2019). Functional Characterization of Clinically-Relevant Rare Variants in ABCG2 Identified in a Gout and Hyperuricemia Cohort. *Cells* 8(4)**,** 363. doi: 10.3390/cells8040363.

Varadi, M., Anyango, S., Deshpande, M., Nair, S., Natassia, C., Yordanova, G., et al. (2022). AlphaFold Protein Structure Database: massively expanding the structural coverage of protein-sequence space with high-accuracy models. *Nucleic Acids Res* 50(D1)**,** D439-D444. doi: 10.1093/nar/gkab1061.

Vuong, H., Che, A., Ravichandran, S., Luke, B.T., Collins, J.R., and Mudunuri, U.S. (2015). AVIA v2.0: annotation, visualization and impact analysis of genomic variants and genes. *Bioinformatics (Oxford, England)* 31(16)**,** 2748-2750. doi: 10.1093/bioinformatics/btv200.

Yang, Y., Muzny, D.M., Reid, J.G., Bainbridge, M.N., Willis, A., Ward, P.A., et al. (2013). Clinical whole-exome sequencing for the diagnosis of mendelian disorders. *N Engl J Med* 369(16)**,** 1502-1511. doi: 10.1056/NEJMoa1306555.

Yoon, J., Cachau, R., David, V.A., Thompson, M., Jung, W., Jee, S.H., et al. (2021). Characterization of a Compound Heterozygous SLC2A9 Mutation That Causes Hypouricemia. *Biomedicines* 9(9)**,** 1172. doi: 10.3390/biomedicines9091172.

Yoon, J., Hwang, Y.S., Lee, M., Sun, J., Cho, H.J., Knapik, L., et al. (2018). TBC1d24-ephrinB2 interaction regulates contact inhibition of locomotion in neural crest cell migration. *Nat Commun* 9(1)**,** 3491. doi: 10.1038/s41467-018-05924-9.
